# Data on master regulators and transcription factor binding sites found by upstream analysis of multi-omics data on methotrexate resistance of colon cancer

**DOI:** 10.1016/j.dib.2016.11.096

**Published:** 2016-12-06

**Authors:** Alexander E. Kel

**Affiliations:** a Institute of Chemical Biology and Fundamental Medicine, SBRAS, Novosibirsk, Russia; b Biosoft.ru, Ltd., Novosibirsk, Russia; c GeneXplain GmbH, D-38302 Wolfenbüttel, Germany

## Abstract

Computational analysis of master regulators through the search for transcription factor binding sites followed by analysis of signal transduction networks of a cell is a new approach of causal analysis of multi-omics data.

This paper contains results on analysis of multi-omics data that include transcriptomics, proteomics and epigenomics data of methotrexate (MTX) resistant colon cancer cell line. The data were used for analysis of mechanisms of resistance and for prediction of potential drug targets and promising compounds for reverting the MTX resistance of these cancer cells. We present all results of the analysis including the lists of identified transcription factors and their binding sites in genome and the list of predicted master regulators – potential drug targets.

This data was generated in the study recently published in the article “Multi-omics “Upstream Analysis” of regulatory genomic regions helps identifying targets against methotrexate resistance of colon cancer” (Kel et al., 2016) [4].

These data are of interest for researchers from the field of multi-omics data analysis and for biologists who are interested in identification of novel drug targets against NTX resistance.

**Specifications Table**

**Table:** 

Subject area	*Biology*
More specific subject area	*Analysis of molecular mechanisms of diseases using NGS, microarrays and novel proteomics technologies*
Type of data	*Table, text file, graph, figure*
How data was acquired	*The data were generated with the help of geneXplain platform version* 3.1 *and 4.0, using databases: TRANSFAC release* 2016.2 *and TRANSPATH* 2016.2.
Data format	*Filtered, analyzed*
Experimental factors	*The samples were used from two states of the cell line of colon cancer HT29: sensitive cells line versus resistant cells.*
Experimental features	*Different omics data were generated in different studies. We extracted the experimental raw data from three repositories: GEO for transcriptomics data, database PRIDE for proteomics data and SRA archives for the epigenomic ChIP-seq data.*
Data source location	*Wolfenbuettel, Germany,* 38302
Data accessibility	*The data is with this article and the initial raw data files are located in the PRIDE database with the project accession number PRIDE:**PRD000369**(**http://www.ebi.ac.uk/pride/archive/projects/PRD000369**); gene expression data is at Gene Expression Omnibus, data entries GEO:**GSE11440**and GEO:**GSE53602*.
	*Processed data and results of data analysis are available in this article and in the publicly accessible section of geneXplain platform at:*http://platform.genexplain.com/bioumlweb/#de=data/Projects/MTX%20resistance/Data/TFs/TF%20sel1%20Transpath%20peptides%20Up%20Upstream%2012%20HT29_protein_context%20viz10all&anonymous=true

**Value of the data**

• Lists of up-regulated and down-regulated genes in MTX resistant cells (Table 1A, 1B, Supplementary material) can help researchers to identify biomarkers of MTX resistance.
• List of predicted transcription factor binding sites (Table. 2, Supplementary material) can be used by other researchers for designing further experiment for experimental validation of gene regulatory mechanisms of MTX resistance.
• List of predicted master regulators (Table. 7, Supplementary material) that can be used for targeted knockout experiments to further investigate the molecular mechanisms of chemotherapy resistance of cancer.

## 1 Data

We here present the results of the analysis of the data of three different omics experiments, namely, transcriptomics, proteomics and epigenomics, that were performed independently in the same type of cell line. After necessary preprocessing of the obtained raw data we performed a special type of computational analysis, which we call “upstream analysis” that helps to integrate these three omics data types and identify master regulators of the methotrexate resistance of colon cancer. We identified master regulators through the search for transcription factor binding sites followed by analysis of signal transduction networks of the cancer cells under study. The found master regulators helped to identify chemical compounds and existing drugs as inhibitors of those master regulators and therefore as potentially helpful for reverting the obtained MTX resistance.

## 2 Experimental design, materials and methods

1) At the first step we analysed the transcriptomics data and compared the MTX resistant and MTX sensitive cells. We revealed differentially expressed genes (DEG) using Limma analysis [1] with the *p*-value cut-off 0.05 (corrected for the multiple testing). Among them, we found 1951 up-regulated genes **(Table 1A Up-regulated genes in_MTXresistant Ensembl.txt,** Supplementary material**)** and 2185 down-regulated genes **(Table 1B Down-regulated genes in_MTXresistant Ensembl.txt,** Supplementary material**)**. Also we extracted a list of genes that did not have significant differences between MTX sensitive and MTX resistant cells (with *p*-value>0.5 and LogFC>−0.01 and <0.01) (**Table 1C, Non-changed genes in_MTXresistant Ensembl.txt,** Supplementary material).
2) At the next step we applied the F-Match algorithm [2] and identified transcription factor binding sites that are overrepresented in promoters of MTX resistant cells in comparison with promoters of MTX sensitive cells. The promoter length was defined from −1000 bp till +100 bp around the transcription start site (TSS). We selected 16 TRANSFAC position weight matrices (PWMs) according to their *p*-value and frequency ratio cut-offs (*P*_value<0.01 & Yes_No_ratio>1.2).
  **(Table 2 Site optimisation summary Up-regulated genes Ensembl FC1.5 sites -1000.100 non-redundant_minSUM_filtered.txt,** Supplementary material**)**
  **Link:**http://platform.genexplain.com/bioumlweb/#de=data/Projects/MTX%20resistance/Data/GSE11440_RAW/Normalized%20(RMA)%20DEGs%20with%20limma/Condition_1%20vs.%20Condition_2/Up-regulated%20genes%20Ensembl%20FC1.5%20sites%20-1000..100%20non-redundant_minSUM/summary%20filtered&anonymous=true.
3) At the next step we applied the CMA algorithm [3] and identified pairs of transcription factor binding sites overrepresented at the promoters of up-regulated genes in MTX resistant cells. We identified 6 pairs of TRANSFAC PWMs the matches of which are clustered in these promoters.
  (see Fig. S2 in [4])
  Link: (also showing the positions of the identified site pairs in the promoters of the up-regulated genes under study)
  http://platform.genexplain.com/bioumlweb/#de=data/Projects/MTX%20resistance/Data/GSE11440_RAW/Normalized%20(RMA)%20DEGs%20with%20limma/Condition_1%20vs.%20Condition_2/Up-regulated%20genes%20Ensembl%20FC1.5%20sites%20-1000..100%20non-redundant_minSUM/CMA%206modules%202sites%20(Up-regulated%20genes%20Ensembl%20FC1.5%20sites%20-1000..100%20non-redundant_minSUM)/Model%20visualization%20on%20Yes%20set&anonymous=true.
  **Table 3 CMA sites in promoters UpFC1.5 track.interval in** Supplementary materials gives genomic coordinates (build GRCh37) of the identified transcription factor binding site pairs in the promoters of the up-regulated genes under study.
4) At the next step we identified peaks of the CDK8 antibody ChIP-seq data in HT29 cell line using the peak calling program MACS [26] (without control and with almost all default parameters, except parameter “Enrichment ratio”, which was set to value 5 in order to achieve higher number of peaks). We identified 29,400 peaks of CDK8 complex binding in the whole human genome. These peaks were mapped to the vicinity of 17,115 genes in human genome (−2000 +2000 around 5′ and 3′ borders of the genes). The information about all these genes with the position of these peaks and the schemas of peak locations in the gene structure is presented here:

Link:

http://platform.genexplain.com/bioumlweb/#de=data/Projects/MTX%20resistance/Data/HT29_ChIP-seq/Track%20genes&anonymous=true.

**Fig. 1:**
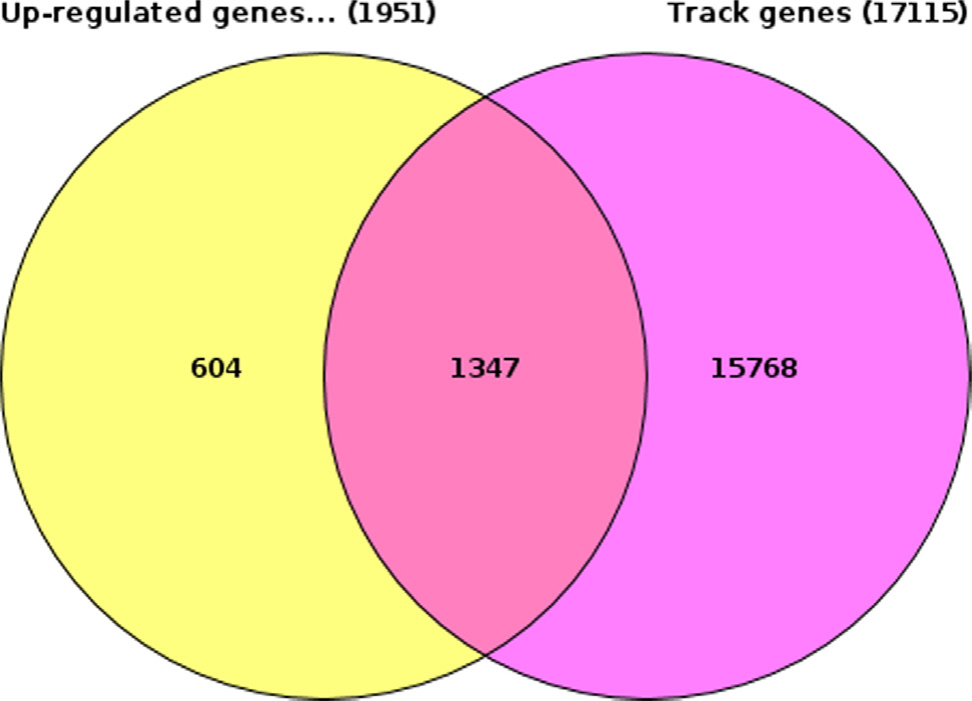
Venn diagram of the overlap between genes associated with at least one peak of the CDK8 antibody ChIP-seq signal and the list of up-regulated in MTX resistant cells.

We retrieved the common genes of this list with the list of upregulated genes in MTX resistant cellsand identified 1347 genes that contain such peaks in their potential regulatory regions (in 5′ regions, in introns, and 3′ regions of the genes). The result of such overlap is shown in Fig. 1 below.

As a result we extracted 710 genomic intervals of 400 bp length each around summits of CDK8 peaks in the up-regulated genes. We consider these intervals as potential MTX resistance enhancers.

**(Table 4 CDK8_400_summit_UpFC1.0_in_MTXresistant.interval,** Supplementary material**).**

**Fig. 2:**
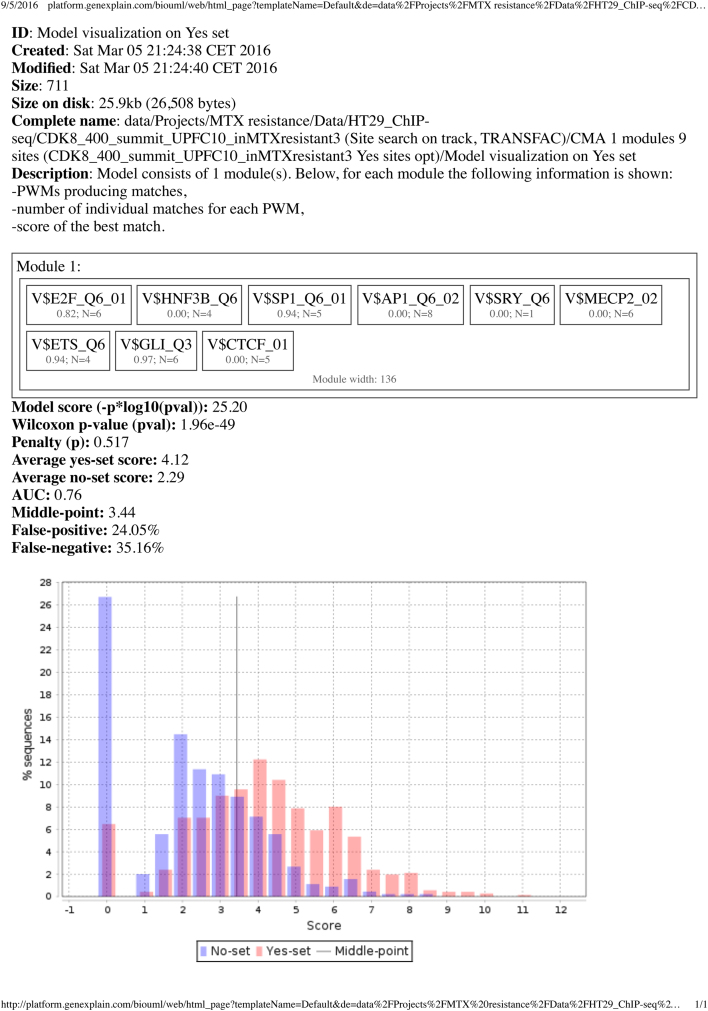
Result of the composite site analysis (CMA) in MTX resistance enhancers. Detailed information of the search algorithm is given. Module 1 represents the list of PWMs that were included by the algorithm into the composite module. Two histograms, red and blue, show the difference of the score of the composite module in the Yes-set (enhancers) and No-set (non-regulated regions of genome).

5) We performed a site frequency analysis (F-Match) and composite site analysis (CMA) in those MTX resistance enhancers in a similar same way as we did in promoters of Up-regulated genes. The results of this analysis is present in Fig. 2 below **(see also the data in Table 5 Site optimization summary_CDK8_400_summit_DnFC1.0.txt,** Supplementary material**).**

6) At the next step we performed the master regulator search as it is described in [2] with a modified algorithm described in the paper [4], using proteomics data as “context proteins”. The proteomics data were matched to the proteins in TRANSPATH database [5]. The list of the TRANSPATH matched proteins found in HT29 cell line is in **Table 6 HT29_colon_cancer_cell_line Ensembl proteins Proteins Transpath peptides a annotated.txt,** Supplementary material.

The master regulator search revealed 48 master-regulator proteins that were either found by the proteomics analysis or whose genes were significantly up-regulated. The list of all revealed master regulators is presented in Table 7 Master regulators from TFs filtered.txt, Supplementary material.

Link:

http://platform.genexplain.com/bioumlweb/#de=data/Projects/MTX%20resistance/Data/TFs/TF%20sel1%20Transpath%20peptides%20Up%20Upstream%2010%20HT29_protein_context%20annotated%20filtered&anonymous=true.
