# Corrigendum to “Data on master regulators and transcription factor binding sites found by upstream analysis of multi-omics data on methotrexate resistance of colon cancer” [Data Brief. 10 (2016) 499–504]

**DOI:** 10.1016/j.dib.2017.06.019

**Published:** 2017-06-17

**Authors:** A.E. Kel

**Affiliations:** a Institute of Chemical Biology and Fundamental Medicine, SBRAS, Novosibirsk, Russia; b Biosoft.ru, Ltd, Novosibirsk, Russia; c GeneXplain GmbH, D-38302 Wolfenbüttel, Germany

The authors regret to report an unfortunate mistake in the Acknowledgements of the paper. It lists several projects that did not provide any financial support for this paper. The only project that provided the financial support for the work described in the paper is the Targeted Program “Research and Development on Priority Directions of Science and Technology in Russia, 2014–2021”, Contract no. 14.604.21.0101, Unique Identifier of the Applied Scientific Project: RFMEFI60414X0101.

Therefore, the following statements are excluded from the Acknowledgements: "The work was partially supported (VP) in the framework of the Russian State Academies of Sciences Fundamental Research Program for 2013–2020. This work was also supported by the following grants of the EU FP7 program: “SysMedIBD” no. 305564, “RESOLVE” no. 305707 and “MIMOMICS” no. 305280."

The correct version of the Acknowledgements is the following: “This work was done with the financial support of Federal Targeted Program “Research and Development on Priority Directions of Development of Science and Technology in Russia, 2014–2020”, Contract no. 14.604.21.0101, Unique Identifier of the Applied Scientific Project: RFMEFI60414X0101.”

The authors would like to apologise for any inconvenience caused.

